# From single crystal neutron diffraction to single crystal diffraction imaging - amazing opportunities and developments

**DOI:** 10.1063/4.0000813

**Published:** 2025-10-27

**Authors:** Christina Hoffmann, James Martin, James Weng, Matthew Krogstad, Ella Schmidt, Reinhard Neder

**Affiliations:** 1 ORNL, Oak Ridge, TN, USA; 2 NCSU, Raleigh, NC, USA; 3 BWXT, Lynchburg, VA, USA; 4 ANL, Argonne, IL, USA; 5 University of Bremen, Bremen, Lower Saxony, Germany; 6 Erlangen University, Erlangen, Bavaria, Germany

## Abstract

At the debut of single crystal neutron diffraction over 80 years ago, it was a courageous undertaking to find diffracting and reflecting intensities in space with a single point detector. Since then, it has been a fantastic journey to watch the methodology progress and it’s impact on discovery of materials and their properties.

Traditional Neutron Bragg intensity measurements integrating discrete “peak above background” counts meanwhile metamorphosed into high resolution diffraction space images that can be effectively combined into complete volumes of reciprocal space.

The structure information between the discrete Bragg peaks in form of intensities from structure modulations that are on step or out of step with the overall long range ordered Bragg structure can be comprehensively visualized and analyzed.

Analysis of materials properties stemming from short range order influences of structural defects, chemical disorder, positional disorder and other structure frustration manifestations in diffuse scattering can be visualized and most recently also analyzed.

This might be a good time to draw attention to an emerging crystalline state at the threshold between fully long range ordered and amorphous or liquid, like plastic crystalline phases. Often, they are present over several degrees Kelvin before a crystal is fully long range ordered. In this state the structured diffuse scattering gives detailed insight of the next neighbor correlations and growing coordination shells. Long range order is forming in the plastic single crystal, which can also be observed.

Data modeling makes use of 3D delta-PDF analysis, showing positive and negative correlations on a “model-free” basis. Recognizing the details that can be discovered in 3D delta-PDF analysis are just beginning to emerge.

This is just one of the exciting developments that mark a transformation from discrete Bragg peak analysis to image processing and modeling which are equally powerful for monochromatic X-ray and polychromatic neutron wavelength resolved diffraction imaging.

A portion of this research used resources at the Spallation Neutron Source, a DOE Office of Science User Facility operated by the Oak Ridge National Laboratory.

